# Model-based PEEP titration versus standard practice in mechanical ventilation: a randomised controlled trial

**DOI:** 10.1186/s13063-019-4035-7

**Published:** 2020-02-01

**Authors:** Kyeong Tae Kim, Sophie Morton, Sarah Howe, Yeong Shiong Chiew, Jennifer L. Knopp, Paul Docherty, Christopher Pretty, Thomas Desaive, Balazs Benyo, Akos Szlavecz, Knut Moeller, Geoffrey M. Shaw, J. Geoffrey Chase

**Affiliations:** 1 Centre for Bioengineering, University of Canterbury, Christchurch, New Zealand; 2 School of Engineering, Monash University, Bandar Sunway, Malaysia; 3 GIGA Cardiovascular Science, University of Liege, Liege, Belgium; 4 Department of Control Engineering and Information, Budapest University of Technology and Economics, Budapest, Hungary; 5 Institute of Technical Medicine (ITeM), HFU Furtwangen University, Villingen-Schwenningen, Germany; 6 Department of Intensive Care, Christchurch Hospital, Christchurch, New Zealand

**Keywords:** ARDS, Mechanical ventilation, Pulmonary mechanics, RCT, PEEP titration, recruitment manoeuvre

## Abstract

**Background:**

Positive end-expiratory pressure (PEEP) at minimum respiratory elastance during mechanical ventilation (MV) in patients with acute respiratory distress syndrome (ARDS) may improve patient care and outcome. The Clinical utilisation of respiratory elastance (CURE) trial is a two-arm, randomised controlled trial (RCT) investigating the performance of PEEP selected at an objective, model-based minimal respiratory system elastance in patients with ARDS.

**Methods and design:**

The CURE RCT compares two groups of patients requiring invasive MV with a partial pressure of arterial oxygen/fraction of inspired oxygen (PaO2/FiO2) ratio ≤ 200; one criterion of the Berlin consensus definition of moderate (≤ 200) or severe (≤ 100) ARDS. All patients are ventilated using pressure controlled (bi-level) ventilation with tidal volume = 6–8 ml/kg. Patients randomised to the control group will have PEEP selected per standard practice (SPV). Patients randomised to the intervention will have PEEP selected based on a minimal elastance using a model-based computerised method. The CURE RCT is a single-centre trial in the intensive care unit (ICU) of Christchurch hospital, New Zealand, with a target sample size of 320 patients over a maximum of 3 years. The primary outcome is the area under the curve (AUC) ratio of arterial blood oxygenation to the fraction of inspired oxygen over time. Secondary outcomes include length of time of MV, ventilator-free days (VFD) up to 28 days, ICU and hospital length of stay, AUC of oxygen saturation (SpO_2_)/FiO_2_ during MV, number of desaturation events (SpO_2_ < 88%), changes in respiratory mechanics and chest x-ray index scores, rescue therapies (prone positioning, nitric oxide use, extracorporeal membrane oxygenation) and hospital and 90-day mortality.

**Discussion:**

The CURE RCT is the first trial comparing significant clinical outcomes in patients with ARDS in whom PEEP is selected at minimum elastance using an objective model-based method able to quantify and consider both inter-patient and intra-patient variability. CURE aims to demonstrate the hypothesized benefit of patient-specific PEEP and attest to the significance of real-time monitoring and decision-support for MV in the critical care environment.

**Trial registration:**

Australian New Zealand Clinical Trial Registry, ACTRN12614001069640. Registered on 22 September 2014.

(https://www.anzctr.org.au/Trial/Registration/TrialReview.aspx?id=366838&isReview=true) The CURE RCT clinical protocol and data usage has been granted by the New Zealand South Regional Ethics Committee (Reference number: 14/STH/132).

## Background

Mechanical ventilation (MV) support is crucial for patients with acute respiratory distress syndrome (ARDS). While there is agreement on the preference for lower tidal volumes [1, 2], there is relatively little consensus on the selection of positive end-expiratory pressure (PEEP) [3–9]. Traditionally, lower PEEP has been used [10, 11], but low PEEP can lead to increases in oxygen desaturation and hypoxaemia [4, 12] and worsening of lung injury, indicated by greater use of rescue therapies and deaths after rescue therapy [9]. High PEEP can increase alveolar recruitment, but can decrease cardiac output and lead to further lung injury due to barotrauma and/or volutrauma [13] or overdistension [4, 9].

PEEP can be optimised to reduce hypoxaemia [6] and intrapulmonary shunting [7] and improve gas exchange [8] and oxygenation [4, 14, 15], by maintaining recruitment of injured or collapsed alveoli [13]. In patients with ARDS, PEEP reduces ventilator-induced lung injury (VILI) [4, 9], increases recruitment [14–16], and reduces inflammatory mediators in plasma and bronchoalveolar lavage fluid [8]. MV strategies combining low tidal volumes with recruitment manoeuvres (RMs) and higher PEEP to prevent VILI have been hypothesized as ideal for lung protection [6, 17]. However, currently, there is still no standardized approach to the selection of this optimal PEEP, or to deciding how often PEEP should be adjusted or recalculated.

Reports from experimental animal trials performed by Carvalho et al., Suarez-Sipmann et al. and Lambermont et al. [18–20] indicate that pigs induced with ARDS experience minimal respiratory elastance at a specific PEEP associated with higher oxygenation, maximum recruitment, and higher functional residual capacity, all without signs of lung overdistension. Equally, it has been proposed that PEEP should be set whereby the lung has minimal respiratory elastance (or maximum compliance), which could be clinically beneficial by balancing the risks of PEEP that is set too low or too high [21–23]. Aside from the work by Suter et al. [21], Pintado et al. also showed that PEEP selection at minimal elastance is beneficial to patients [22]. Despite some consistent findings, the application of minimal elastance PEEP selection remains limited and hindered by the lack of an objective, reliable, and easy-to-use method to determine elastance at the bedside in real time.

Chiew et al. showed the potential benefit of minimal-elastance PEEP selection in a pilot study [23, 24]. Following the study, a phase-2 randomised controlled trial (RCT) was designed to assess mechanical ventilation at minimal elastance PEEP in patients with ARDS versus standard practice of care in a single-centre hospital. In particular, patient-specific respiratory system elastance and corresponding minimal elastance PEEP is determined using a validated model-based method and computer software [25]. This trial uses real-time-identified patient-specific respiratory system elastance, and thus the trial is named the Clinical utilisation of respiratory elastance (CURE) RCT. This manuscript presents the detailed clinical protocol for the phase-2 CURE RCT. This trial is registered with the Australian New Zealand Clinical Trial Registry (ANZCTR): ACTRN12613001006730.

## Methods and trial design

### Study design and setting

The CURE RCT is a two-arm RCT comparing model-based mechanical ventilation (MBV) with current standard practice mechanical ventilation (SPV) in patients with a ratio of partial pressure of arterial blood oxygen (PaO2)/fraction of inspired oxygen (FiO2) (P/F ratio) ≤ 200. It is to be conducted in a single-centre hospital intensive care unit (ICU) at Christchurch Hospital in Christchurch, New Zealand.

The primary objective is to assess the impact of model-based ventilation in PEEP selection (MBV) therapy on clinically significant patient outcomes and patient-centred quality of care metrics. The other objectives of this study include (1) to provide the knowledge and methods to make care more patient-specific and timely to optimise treatment and improve outcomes in a large cohort of critically ill patients and (2) to improve the understanding of the pathophysiological basis of critical illness through what we learn about the hourly and daily evolution of lung injury in terms of patient-specific elastance and response to care through this study.

The primary outcome of this study is the area under the curve (AUC) of PaO2/FiO2 over the period of mechanical ventilation. Secondary outcomes include length of time of MV (LoMV), ventilator-free days (VFD) up to 28 days, ICU and hospital length of stay (LoS), AUC of SpO_2_ (oxygen saturation)/FiO_2_ during MV, number of desaturation events (frequency and fraction of time SpO_2_ < 88%), changes in respiratory mechanics and chest x-ray index scores, rescue therapies (prone positioning, nitric oxide use, extracorporeal membrane oxygenation (ECMO)) and hospital and 90-day mortality. These outcomes and their corresponding four levels of specification based on Zarin et al., 2011 is shown in Table 1. The secondary analysis includes comparison of the means of LoMV, VFD, hospital and ICU LoS, 90-day mortality, chest x-ray index scores and rescue therapies used.

**Table 1:** Four levels of specification in primary and secondary outcomes

Level 1 Domain	Oxygenation		MV		Length of stay			Other	
Level 2 Specific domain	AUC P/F ratio	AUC Spo2/FiO2	Length of MV	Ventilator free days (VFD) up to 28 days	Hospital	ICU	90-Day mortality	Chest x-ray index scores	Rescue therapies
Level 3 Specific metric	Difference in AUC		Number of days	28 days – days of MV	Number of days		Time to event	Index scores over period of MV	Number of therapies used
Level 4 Method of aggregation	Comparisons of the of means of AUC		Comparisons of the means of number of days	Comparisons of the means of VFD	Comparisons of the means of length of stay		Comparison on number of 90-day mortality	Comparison of index scores over time	Comparison of rescue therapies used.

*P/F* partial pressure of arterial oxygen/fraction of inspired oxygen, *SPO*_*2*_ oxygen saturation, *MV* mechanical ventilation, *FiO*_*2*_ fraction of inspired oxygen

A difference in primary and secondary outcomes will show the impact of MBV compared to SPV. No difference would show that enhanced, model-based metrics of patient-specific condition have no effect on patient-centred or clinical outcomes. Either outcome will yield clinical guidance.

### Two-arm RCT

Eligible patients are randomised to either the model-based intervention group (MBV) or the control group (SPV). Both groups will have designated computer software to monitor their breathing [25]. The software uses real-time measurements of pressure and flow from the ventilator to objectively calculate the patient-specific and breath-specific respiratory system elastance for every breath [25].

Participants on MBV will undergo recruitment manoeuvres (RM), an initial maximum recruitment manoeuvre (RM_Max_) or subsequent PEEP adjustment and monitoring procedure (PUMP) mini recruitment manoeuvres. The respiratory elastance at each PEEP step during these protocolised RMs is calculated and recorded. The software will recommend a patient-specific minimal-elastance PEEP to the clinicians in setting ventilator PEEP. Patients on SPV will have PEEP selected using current clinical practice without the aid of the software, but all breaths will be analysed and elastance recorded; clinical staff will be blinded to these data.

#### Adherence to the intervention

Patients recruited into this study will be under constant supervision in the ICU. However, their outcomes will be measured based on the intention-to-treat principle, taking into account protocol variations, which naturally occur. These variations will be reported to the primary investigator at the earliest opportunity and followed up. There will be detailed training on the use of CURE equipment and on the protocol, to allow adherence to trial.

#### Protocol amendments

This trial is based on the intention-to-treat principle. Thus, protocol amendments may be required to ensure patient safety and outcomes, and the primary investigators will instigate protocol amendments if necessary. The amendments will be reviewed by the data monitoring committee (DMC) to warrant patient safety and outcomes. The DMC may also refer protocol amendments based on outcomes of the interim analysis reports. Finally, if participant enrolment is slow, the protocol may be amended to allow faster recruitment.

#### Concomitant care and intervention

The trial involves critically ill participants who are mechanically ventilated. Thus, it is likely and acceptable for participants to be receiving medication related to any other concomitant comorbid conditions while participating in the CURE RCT.

Participants in this study will not be concomitant to another study that would affect the results of this study. Participants will not be co-enrolled in another study that uses different oxygenation settings, recruitment manoeuvre procedures or anything that may affect the outcomes of this study.

### Eligibility criteria

The following are the CURE RCT inclusion, exclusion and P/F ratio criteria.

#### Inclusion criteria

The inclusion criteria are:

1. P/F ratio ≤ 200
  i. on any level of PEEP or FiO_2,_ or
  ii. P/F ratio ≤ 200 on FiO_2_ = 50% and PEEP = 5 (see “P/F ratio criteria”)

#### Exclusion criteria

The exclusion criteria are:

1. P/F ratio > 300 on any level of PEEP or FiO_2_
2. P/F ratio > 200 on FiO_2_ = 50% and PEEP = 5
3. Ventilated > 48 h (including time spent in another hospital)
4. Not expected to be ventilated for another 48 h
5. Age < 16 years
6. Any medical condition associated with a clinical suspicion of raised intracranial pressure and/or measured intracranial pressure ≥ 20 mmHg
7. High spinal cord injury with loss of motor function and/or significant weakness due to neurological disease
8. Barotrauma (pneumothorax, pneumomediastinum, subcutaneous emphysema or any intercostal catheter for the treatment of air leak)
9. Asthma as the primary presenting condition or history of significant chronic obstructive pulmonary disease
10. Moribund and/or not expected to survive for > 72 h
11. Agreed limitations of care due to co-morbidities, or not expected to survive 90 days
12. Lack of clinical equipoise as determined by ICU medical staff managing the patient (for example, if the patient has unremarkable findings on chest x-ray, with the possibility of thrombotic or fat pulmonary emboli)
13. Previous enrolment in the CURE RCT

#### P/F ratio criteria

The P/F ratio criteria are:

1. If the P/F ratio is ≤ 200, the patient is eligible for enrolment
2. If 200 < P/F ratio ≤ 300, set FiO_2_ = 50% and PEEP = 5 cmH_2_O and repeat the analysis of arterial blood gases (ABG) within 10 min of the change to measure the new P/F ratio
  - If the new P/F ratio is ≤ 200, the patient is eligible for enrolment
  - If the new P/F ratio is > 200, the patient is not eligible for enrolment and, if appropriate, will be re-screened at a later time

This trial will recruit patients who have a P/F ratio ≤ 200, a criterion in the definition of severe to moderate ARDS as defined by the ARDS Definition Task Force in the Berlin definition [26]. They will be eligible if their P/F ratio is ≤ 200 on any level of PEEP and FiO_2_. Those patients with a 200 < P/F ratio ≤ 300 will be placed on PEEP = 5 cmH_2_O, and FiO_2_ = 50%. If a subsequent P/F ratio is ≤ 200 they will also become eligible (see Additional file 1). The P/F ratio measured at FiO2 of 50% and PEEP of 5cmH_2_O is based on Villar et al. 2013 [27].

### Consent, compliance and withdrawal

#### Consent procedure

First, it is important to note that standard ventilation practice may include recruitment manoeuvres to increase lung recruitment and oxygenation. However, these clinical practices are widely variable and often not standardised. The recruitment techniques used to improve oxygenation and mechanics of ventilation in the intervention and control arms of this study are within the scope of standard ICU clinical practice. The protocols used will standardise these existing interventions to recruit lung volume and titrate PEEP.

Study participants will be unable to consent to participation in this study prior to enrolment as they will be sedated and mechanically ventilated. It is also equally important to randomise participants to either arm of the RCT at the commencement of MV to ensure a fair comparison. Patients who have been ventilated ≤ 48 h are eligible for the CURE RCT. Given this time frame, the CURE RCT will recruit patients once family consent is obtained. However, if the treating clinician firmly believes a recruitment manoeuvre is in the best interests of the patient, and no family is available for consent, the participant will be enrolled and randomised and the appropriate protocolised recruitment manoeuvre will follow. In this case, delayed consent is obtained as early as possible. Once the participant recovers from their condition and is discharged from the ICU, we will seek their informed consent.

In cases where the family cannot attend the hospital to sign a statement of assent, their opinion will be obtained by telephone in the first instance. Information about the study will either be made available by emailing them the information sheet and contacting them later by telephone, or the information sheet will be read to them over the telephone. The telephone conversation(s) and their opinions will be documented in the patient’s medical record. As soon as the family is able to attend the hospital, they will be asked to sign the statement. If the family are not able to sign a statement during the patient’s time in the ICU, they have the option of printing out the statement, signing it, and mailing, emailing or faxing it back. The sample study information and consent forms can be seen in Additional file 2.

#### Withdrawal of consent

If the participant’s family, relative or friend does not agree to their continued participation, they will be withdrawn from the study and we will seek agreement from them to use information related to mechanical ventilation that was collected up until that point.

If a participant chooses to withdraw from the trial, we also will seek agreement to use information related to mechanical ventilation that was collected up until that point. If they do not agree, then all study information obtained will be destroyed.

### Randomisation and blinding

Participants will be block-randomised, with block sizes generated using a randomisation programme. The programme will randomly assign patients into either a control group or intervention group through a random block size (the block size is 4, 6, 8 or 10 patients). Eligible and consented patients will be block-randomised in a ratio of 1:1. No effort will be made to stratify the subgroups considered in the secondary analyses. By the nature of the intervention, CURE cannot be double-blinded. Un-blinding is not applicable due to the nature and setting of the intervention.

All patient data collected are de-identified using a single patient numbering system. Patients are assigned to a study number to ensure no bias in the results. This system will be a simple incrementing scheme, such that patients randomised into the CURE trial are identified as study-001, study-002, etc.

### Ventilation settings, oxygenation, and patient positioning

#### Tidal volumes (V_T_) and driving pressures (DP) during MV

The V_T_ is adjusted to 6–8 ml/kg per ideal body weight (IBW), and the maximum minute ventilation (V_Emax_) to ≤ 0.2 L/kg/min. The IBW is measured using the patient’s height and look-up table at the bedside or is calculated using the formulae:

1 $$ \mathrm{Men}:50+0.91\times \left(\mathrm{height}\ \left[\mathrm{cm}\right]-152.4\right)\ \mathrm{kg} $$

2 $$ \mathrm{Women}:45.5+0.91\times \left(\mathrm{height}\ \left[\mathrm{cm}\right]-152.4\right)\ \mathrm{kg} $$

The DP is the plateau pressure (P_Plat_) minus the PEEP. In patients with very severe ARDS the adjustment of V_T_ to 6–8 ml/kg IBW may be injurious if the DP is higher than 15 cmH_2_O. A DP ≤ 15 cmH_2_O was associated with better patient survival when assessed using a multilevel mediation analysis of 3562 patients in nine RCTs of ARDS [28]. Therefore, the DP will be limited to ≤ 15 cmH_2_O at all times. In addition, during spontaneous ventilation, pressure support will be limited to ≤ 15 cmH_2_O.

The ventilation rate is set between 12 and 20 breaths per minute. The aim is to keep the plateau pressure P_plat_ ≤ 30 cmH_2_O. If necessary, V_T_ may be reduced to as low as 4 ml/kg and the respiratory rate (RR) kept at ≤ 30 breaths per minute. CO_2_ will frequently rise in severe lung injury (permissive hypercapnia) when patients are mechanically ventilated within these guidelines. However, if CO_2_ is ≥ 80 mmHg or increased by ≥ 50% in the previous 4 h, the intensive care specialist on duty will be notified, and they may choose to deviate from these guidelines.

#### Ventilation mode

All patients enrolled are to be ventilated using a pressure-controlled mode, for example, the Bi-Level ventilation mode on the Puritan Bennett PB840 ventilator (Covidien, Boulder, CO, USA) or PC-SIMV+ on the Dräger Evita® Infinity® V500. Patients will be ventilated using Bi-Level/PC-SIMV+ mode, which allows unrestricted spontaneous breathing efforts to lessen ventilator dyssynchrony. However, during any recruitment manoeuvre procedures, synchronized intermittent mandatory ventilation (SIMV) with pressure-controlled (PC) ventilation is used and returned to original mode afterwards. Should patients already be ventilated using a ventilator incompatible with the CURE computer system, they will have their ventilator changed to a compatible ventilator for the trial. Patients will be transitioned to assisted spontaneous breathing (ASB) if they meet the weaning criteria (see “Weaning”).

In severe ventilator dyssynchrony, a very high respiratory drive may result in sub-atmospheric circuit pressures and risk of aspiration of gastric contents around the endotracheal cuff. If a participant has a high respiratory drive on Bi-Level/PC-SIMV+ ventilation, producing a fall in airway pressure during inspiration, muscle relaxants will be considered to facilitate controlled breathing. However, if the clinician feels the participant may benefit from breathing spontaneously, transition to ASB may be made if they substantially meet the weaning criteria (see “Weaning”).

However, spontaneous breathing efforts may mask high trans-pleural pressures and produce high levels of regional lung strain. Oesophageal pressure will not be measured during this trial. If the treating clinician is concerned about patient self-inflicted lung injury (P-SILI) [29, 30], they will consider using muscle relaxants to control ventilation.

Patients will not undergo any procedures using a cough-assist machine prior to weaning and transitioning to spontaneous breathing. However, the treating clinician may use a cough-assist machine to aid secretion removal (during spontaneous breathing) if they believe it is in the patient’s best interests. Finally, in any circumstances where the patient is planned to be temporarily disconnected from the ventilator, their endotracheal tube will be clamped to prevent de-recruitment.

#### SpO_2_ targets

To ensure a fair comparison, all CURE study participants will have inspired oxygen levels titrated to achieve the following pulse oximetry saturations:

i. SpO_2_ = 93–95% if FiO_2_ is less than 60%
ii. SpO_2_ = 90–92% if FiO_2_ is greater than or equal to 60%

The aim is to spend greater than or equal to 90% of time in the target range. The FiO_2_ should only be increased above 21% if these targets are not met, using 5% increments starting with a FiO_2_ = 25%. There is natural variability in SpO_2_. To avoid toggling between two FiO_2_ levels, 10 min of settling time will be allowed before changing the FiO_2_. The best FiO_2_ is chosen to keep the saturation within the specified targets ranges over 90% of the time.

#### Patient position, turning and prone positioning

Patients are kept at 30° head up whenever possible. This position maximises recruitment of the lung and may reduce the risk of aspiration. Wherever possible, patients should be rolled from supine to right-side down, back to supine, then to left-side down. This turning of patients is ideally performed every 3 h.

Transient hypoxaemia frequently occurs after a patient has been turned and may be worse if there is inadequate PEEP. Hypoxaemia may also become more severe if participants are rolled from left-side down to right-side down due to cyclical de-recruitment of the non-dependent lung and re-recruitment of the dependent lung. This cyclical de-recruitment of the lung has the potential to contribute to VILI. Thus, patients with severe lung injury may be very intolerant of being turned. In some instances, the lungs may need to be re-recruited. If desaturation does occur, this will be recorded as a serious adverse event (SAE).

Prone positioning of patients may be considered if the P/F ratio is ≤ 100 and FiO_2_ ≥ 60%. Patients randomised to the intervention arm (MBV) may still undergo a protocolised recruitment manoeuvre. For patients in the standard practice ventilation arm (SPV) a staircase recruitment manoeuvre is left to clinical judgement.

### ABG recordings

The primary outcome of this trial is the AUC of the P/F ratio. For this reason, mandatory daily ABG recordings are performed for up to 10 days after enrolment. ABGs are taken around 0600 hours and 1800 hours. The ABGs are also acquired within 60 min of any recruitment manoeuvre procedure and 30–60 min after the recruitment manoeuvre procedure. The added ABGs from RM procedures will be used in secondary analysis, but will be omitted during primary analysis to ensure the same number of data points per day for all patients.

### Duration of the intervention

**Fig. 1:**
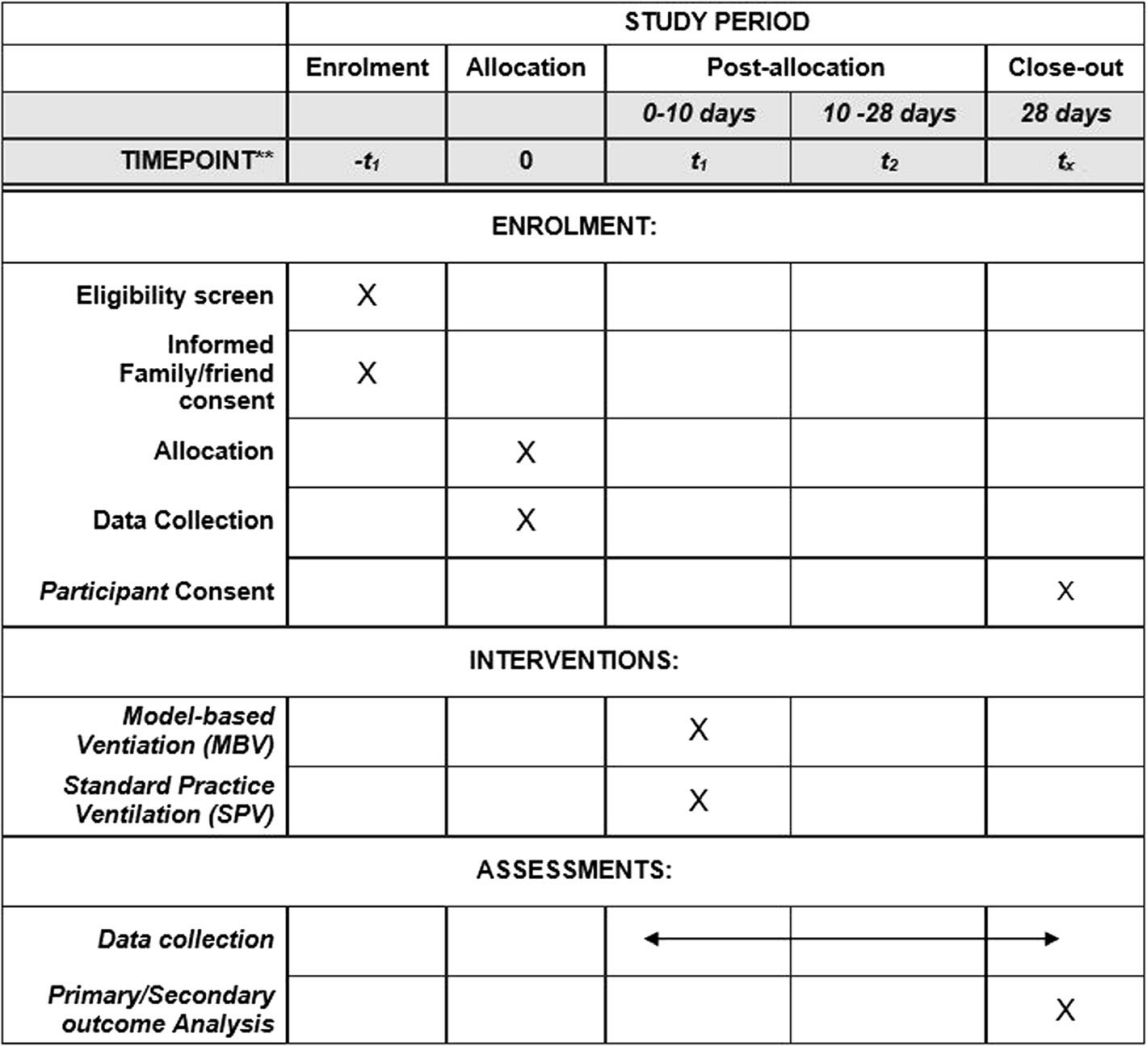
Trial assessment schedule

Patients randomised to the model-based ventilation (MBV) cohort will remain in the protocol up to 10 days. Thereafter, they will receive the same care as participants assigned to standard practice ventilation (SPV). However, if participants have been extubated, but then require intubation and re-ventilation at any time within 10 days of enrolment, they will return to the original assigned protocol (MBV or SPV). All patients will receive standard care beyond 10 days of enrolment, and their data, including ABG recordings, will continue to be collected for up to 28 days. This intervention schedule is shown in Fig. 1.

### General procedures

#### Procedures for the control group (SPV)

The procedures are as follows:

1. PEEP is selected as per standard practice.
2. The decision to carry out a staircase recruitment manoeuvre will be based on clinical judgement. The protocol for performing the staircase recruitment manoeuvre is explained in “Recruitment manoeuvres (RM)”.
3. ABGs will be taken twice daily.
4. Ventilator data are collected continuously until the ventilator is disconnected.

#### Procedures for the intervention group (MBV)

The procedures are as follows:

1. For patients included in the MBV (intervention) group, the PEEP and MV will be guided by clinicians using bedside computers, while maintaining V_T_ and FiO_2_.
2. Patients randomised to MBV will undergo protocolised recruitment manoeuvres (RM)
  i. Before the RM, the patient should be sedated and paralysed, if required, with muscle relaxants to prevent spontaneous breathing efforts.
  ii. The first RM used is a maximum recruitment manoeuvre (RM_Max_). This is done at the beginning of the trial by clinicians and only repeated if clinically indicated.
  iii. The PEEP adjustment and monitoring procedure is referred to as a “PUMP”, whereby the PEEP is adjusted − 4 cmH_2_O to + 4 cmH_2_O of the current PEEP setting. The PUMP may be performed by ICU staff trained in this technique.
3. The participant will no longer undergo a PUMP when:
  - FiO_2_ ≤ 35%,
    i. And they have fully transitioned to spontaneous breathing
    ii. And the PaO_2_ was ≥ 60 mmHg for the last 24 h, or
  - After 10 days from study enrolment, or
  - At the discretion of the clinician, for example:
    iii. When they have a new neurological condition.
    iv. They are awake and breathing normally without evidence of respiratory distress, and where sedation (with or without paralysis) is not considered to be in their best interests.
4. ABGs will be recorded twice daily and before and after any recruitment procedure.
5. Data will be collected continuously until the patient is disconnected from the ventilator.

## Recruitment manoeuvres (RM)

Patients enrolled in this study will undergo RMs. The RMs are only carried out by senior medical staff or senior trainees familiar with this technique. RM_Max_ and PUMPs are for participants randomised to the MBV protocol arm only. Patients assigned to the SPV arm may undergo a staircase RM (SRM) at the discretion of the treating clinician according to standard practice. (see “Standard practice staircase recruitment manoeuvre”).

All RMs will be performed in SIMV pressure controlled (PC) ventilation mode. The peak inspiratory pressure (Pi) is set to achieve a V_T_ of 6–8 ml/kg IBW. Preferably, V_T_ should result in DP ≤ 15 cmH_2_O above PEEP.

Before and after each RM, ABGs (pH, PaCO_2_, PaO_2_, HCO_3_), SpO_2_, end-tidal CO_2_ partial pressure (ETCO_2_), FiO_2_, PEEP, RR), and V_T_ will be recorded. In addition, during the RM_max_ (model-based ventilation arm) or (standard practice ventilation (SRM) arm, at each PEEP increment, Heart rate, rhythm, mean arterial pressure (MAP), SpO_2_, FiO_2_, V_T_, RR, ETCO_2_ and rates of use of vasoactive drugs will be recorded. This data will be valuable in assessing the safety of the RM_Max_, PUMP and SRM.

In many cases, where the lung stiffness, or respiratory elastance (E) is high, it will not be possible to deliver a V_T_ of 6 ml/kg IBW. Furthermore, during the RM, the delivered V_T_ may fall further as the elastance increases. As a result, it may be necessary to increase the respiratory rate to accommodate the reduction in minute ventilation. For example, if E is > 40 cmH_2_O/L (or compliance < 25 ml/cmH_2_O) in a patient weighing 58 kg (IBW), (normal range 15–20 cmH_2_O/L), the V_T_ will be < 6 ml/kg (< 350 ml) when the driving pressure is 15 cmH_2_O.

It is important that oxygenation targets in both arms are carefully followed to ensure a fair comparison between them. The SpO_2_ will be kept in the target range prior to any RM. This approach allows small decreases in oxygenation to be detected during the decremental PEEP phase of the recruitment manoeuvre, while also providing a sufficient buffer in the event of significant de-saturation due to ventilation perfusion (V/Q) mismatch. V/Q mismatch increases with higher airway pressures when pulmonary arterial blood is shunted away from the pulmonary capillaries by-passing aerated regions of the lung.

### RM checklist

Before performing any RM, the following criteria are considered. Any RM must be delayed until these conditions are corrected in the consideration of the following at-risk patient conditions:

1. Haemodynamic instability (e.g. ongoing haemorrhage).
2. Not optimally resuscitated with fluids (e.g. stable blood pressure, but pulse pressure variation ≥ 12% because of inadequate left ventricular preload)? This is only applicable in the absence of spontaneous breathing.
3. Evidence of barotrauma since enrolment?
  - If there is new barotrauma, RMs must not be attempted and the participant will be withdrawn from trial. They will continue to be observed and followed up. A SAE will be reported.

### RM preparation steps

Once the RM checklist conditions are met, the patient can be prepared for a RM by ensuring:

1. There is a reliable arterial line.
2. The patient is supine, 15–30° head up.
3. The endotracheal tube (ETT) cuff is inflated to 45 cmH_2_O (RM_Max_ or SRM) or 35–40 cmH_2_O (PUMP) to ensure there is no leak at maximum airway pressures. The ETT cuff is deflated to less than 30 cmH_2_O at the end of the RM procedures.
4. The peak pressure alarm is set to 45 cmH_2_O (RM_Max_, PUMP, SRM)
5. SpO_2_ is in the target range (FiO_2_ < 60%: 93–95% or FiO_2_ ≥ 60%: 90–92%), and ABGs have been recorded within the last 60 min.
6. If the patient is not on vasoactive drugs, intravenous (i.v.) adrenaline (or other suitable vasoactive drug) is available in the event of hypotension.

### RM termination

RMs should be terminated if at any time during the RM any of the following changes persist for more than 3 min:

1. Desaturation, with SpO_2_ < 88%.
2. New bradycardia (heart rate < 60 beats per minute) or,
3. New tachycardia (heart > 140 beats per minute) or,
4. New arrhythmia leading to new bradycardia (2) or new tachycardia (3) or,
5. New hypotension (reduction in MAP by 40% or MAP < 60 mmHg).

This RM termination criteria applies to all RM procedures in both arms.

### RM_Max_; MBV

The RM_Max_ is a computer-guided staircase RM procedure in the MBV intervention arm. This method is designed to safely increase the inspiratory pressure to a maximum airway pressure of 40–43 cmH_2_O, with DP limited to 15 cmH_2_O, and maximum PEEP limited to 25–28 cmH_2_O. The RM_Max_ is guided by the CURE software using a validated model-based method, which estimates elastance to determine the optimal PEEP [23, 31].

The RM_Max_ is carried out by intensive care specialists or senior trainees familiar with this technique. This procedure is only carried out during working hours (0800–1800 hours), but preferably within 4–6 h of enrolment. However, for patients enrolled overnight, unless there are compelling reasons to carry out an RM_Max_, this procedure may be delayed till the following morning (0800 hours).

Contra-indicated preconditions to an RM_Max_ are excluded using the RM checklist. If it is safe to proceed, the patient is prepared for the RM_Max_.

The following instructions are given to the clinician:

1. Adjust oxygenation to meet the target range.
2. Titrate sedation so the patient is not verbally responsive and has loss of their eyelash reflex. Use fentanyl or morphine increments with propofol to provide a “balanced” deeper sedation level. Give rocuronium 1.0–1.5 mg/kg through a reliable i.v. line; ensure the line is flushed.
3. Set the ventilator to SIMV-PC (pressure control) mode.
4. Set peak inspiratory pressure (Pi) to 15 cmH_2_O above PEEP.
5. Ensure the initial PEEP is ≤ 15 cmH_2_O.
6. Start maximum recruitment on the CURE soft programme.
7. Follow the steps of the CURE soft protocol: during the RM_Max_, increase PEEP in steps of 4 cmH_2_O above the baseline PEEP level until Pi reaches 40–43 cmH_2_O or PEEP 25–28 cmH_2_O. Then reduce PEEP in 4 cmH_2_O decrements until the original starting PEEP is reached. Adjust FiO_2_ throughout the procedure to keep SpO_2_ ≥ 90%.
8. Once PEEP has been returned to the initial setting, perform a second RM_max_ using the same method in point 7. The RM_Max_ is carried out twice. During the first RM_Max_ the elastance changes in PEEP level are more variable and therefore less predictable. The non-recruited lung is highly heterogeneous with regions of collapse and consolidation. The first RM_Max_ is used to recruit these de-recruited regions. The second manoeuvre is used to estimate optimal PEEP from repeated estimates of elastance changes during decremental PEEP titration.
9. The CURE soft programme will recommend an optimal PEEP at the end of the second RM_Max_. You may either accept this computerised recommendation, or reject it (with a reason) if you feel the new PEEP level is inappropriate. If rejected, record your reason(s) on the programme.
10. Return the patient to the previous ventilation mode.
11. Adjust V_T_ to ≤ 6–8 ml/kg IBW. If the plateau pressure is > 30 cmH_2_O, adjust the V_T_ down to 4–6 ml/kg IBW and tolerate permissive hypercapnia. Also ensure that the DP remains at ≤ 15 cmH_2_O. You may increase the respiratory rate up to 30 breaths/min provided there is no significant auto-PEEP causing breath stacking.
12. Reduce the ETT cuff pressure to the previous level and re-set ventilator alarms to previous settings.
13. Record ABGs 30–60 mins after conclusion of the procedure.

### Repeating the RM_Max_

The RM_Max_ may be repeated only when the following conditions are met:

1. If there is a significant change in the participant’s condition, e.g. new severe hypoxaemia (SpO_2_ < 90% and FiO_2_ ≥ 60%; P/F ~ 100) and
2. Patient conditions for which lung recruitment is contraindicated are excluded (e.g. endobronchial intubation, mucous plugging, pneumothorax etc) and
3. Analgesia and sedation and patient position have been optimised (consider small changes to respiratory rate, V_T_ and pressure support, or a rocuronium infusion) and
4. The PUMP fails to improve oxygenation

### PUMP: PEEP adjustment and monitoring procedure

PUMP is a regular mini-recruitment manoeuvre procedure designed to adjust PEEP based on patient-specific changes in condition. This mini-RM is also guided by CURE software and moves between ±4 cmH_2_O from the current PEEP. PUMP should be performed twice daily during normal working hours (0800–1800 hours) or at any other time if lung de-recruitment is considered to be the likely cause of new desaturation. The Pi will be left the same as in the current ventilator settings. To ensure a PUMP can be safely carried out, the RM checklist and preparation steps are to be followed. If the checklist preconditions are met, the PUMP may be carried out.

The following instructions are given to the clinician:

1. Titrate sedation so the patient is not verbally responsive and has loss of their eyelash reflex. Use fentanyl or morphine increments with propofol to provide a “balanced” deeper sedation level. Give rocuronium 0.5–1.0 mg/kg through a reliable i.v. line; ensure the line is flushed.
2. Set the ventilator to SIMV-PC mode with the appropriate aforementioned settings.
3. Reduce the PEEP to 4 cmH_2_O less than the current PEEP setting. The CURE software cannot estimate an optimal PEEP that is lower than the current PEEP setting.
4. Start PUMP on the CURE soft programme.
5. Follow the steps of the CURE soft PUMP protocol. During the PUMP procedure, increase PEEP in two steps of 4 cmH_2_O. Then decrease PEEP in two steps of 4 cmH_2_O (you may need to adjust the FiO_2_ to keep the SpO_2_ ≥ 90%).
6. Once you have returned PEEP to the starting PEEP level (initial PEEP − 4 cmH_2_O), perform a second PUMP using the same method described in point 5.
7. The CURE soft programme will recommend a new PEEP at the end of the second PUMP. You may either accept this computerised recommendation, or reject it (with a reason) if you feel the new PEEP level is inappropriate. If rejected, record your reason(s) on the programme.
8. Return the patient to the previous ventilation mode.
9. Adjust to V_T_ ≤ 6–8 ml/kg IBW. If the plateau pressure is > 30 cmH_2_O, adjust the V_T_ down to 4–6 ml/kg IBW and tolerate permissive hypercapnia. Also ensure that the DP remains ≤15 cmH_2_O. You may increase the respiratory rate up to 30 breaths/min provided there is no significant auto-PEEP causing breath stacking.
10. Reduce the ETT cuff pressure to the previous level and re-set ventilator alarms to previous settings.
11. Take an ABG 30 to 60 mins following the conclusion of the procedure

### Standard practice SRM

Participants assigned to SPV have PEEP determined using clinical judgement, as per local unit standard care. However, if oxygen requirements are high or have recently increased e.g. an FiO_2_ ≥ 50% to keep SpO_2_ in the target range of 93–95%, the following should be considered if the treating clinician is intending to carry out an SRM:

1. Patient conditions for which lung recruitment are contraindicated are excluded (e.g. endobronchial intubation, mucous plugging, pneumothorax etc.)
2. Analgesia and sedation, and patient position have been optimised
3. Small changes to respiratory rate, V_T_, pressure support or neuromuscular blockade to optimise mechanical ventilation

If the oxygenation does not improve with the aforementioned interventions, then PEEP may be increased in increments of 2 cmH_2_O. If the PEEP is ≥ 15 cmH_2_O and FiO_2_ is ≥ 60%, (P/F ~ 100) in spite of addressing the aforementioned points, a staircase recruitment manoeuvre (SRM) may be considered if the clinician feels this is in the best interests of the patient.

The SRM procedure does not utilise the CURE software to perform recruitment and therefore the software will not guide the user, nor make any PEEP suggestions. The software will still record airway pressure and flow through this procedure.

To ensure a SRM, can be safely carried out, the RM checklist and preparation steps are to be followed. If the checklist preconditions are met, the SRM may be carried out (note, the SRM procedure does not utilise CURE software to perform recruitment).

The following instructions are given to the clinician:

1. Titrate sedation so the patient is not verbally responsive and has loss of their eyelash reflex. Use fentanyl or morphine increments with propofol to provide a “balanced” deeper sedation level. Give rocuronium 1.0–1.5 mg/kg through a reliable i.v. line; ensure the line is flushed.
2. Set the ventilator to SIMV-PC mode.
3. Set Pi to 15 cmH_2_O above PEEP.
4. Ensure the initial PEEP is ≤ 15 cmH_2_O. If PEEP is set at ≤ 15 cmH_2_O, the corresponding plateau pressure will not exceed 30 cmH_2_O.
5. Increase PEEP in a stepwise manner every minute in steps of 4 cmH_2_O to a achieve Pi of 40–43 cmH_2_O.
6. Reduce PEEP to 24, and then by decrements of 2 cmH_2_O, every 2 min, until the SpO_2_ begins to fall by no less than 2% of the maximum observed. Hold PEEP at this level and then increase PEEP to the maximum that was previously used for 1 min before returning to a PEEP level 2 cmH_2_O above the level when the SpO_2_ was first noted to have fallen (the decrements in 2 cmH_2_O will allow a PEEP selection between 16 and 24 cmH_2_O, which is within the high PEEP protocol from the ARDS Clinical Network study of high versus low PEEP [4].
7. If there is no desaturation during the decremental phase of the SRM, reduce PEEP to 16 cmH_2_O; no further changes in PEEP are required.
8. Return the patient to the previous ventilation mode.
9. Adjust the DP to ≤ 15 cmH_2_O to give a V_T_ ≤ 6–8 ml/kg IBW. If the plateau pressure is > 30 cmH_2_O, adjust the DP so that V_T_ is to 4–6 ml/kg IBW and tolerate permissive hypercapnia. You may increase the respiratory rate up to 30 breaths/min provided there is no significant auto-PEEP causing breath stacking.
10. Reduce the ETT cuff pressure to the previous level and re-set ventilator alarms to previous settings. Record ABGs 30–60 mins after the conclusion of the procedure.

## Ventilator dyssynchrony

Ventilator dyssynchrony occurs when a patient’s spontaneous respiratory efforts are not synchronised with the ventilator. This commonly causes agitation and respiratory distress; often described as “fighting the ventilator”. Dyssynchrony should be considered in patients with increased respiratory efforts, unexplained agitation, tachycardia, or sweating. Ventilator wave forms can be used to identify dyssynchrony.

**Fig. 2:**
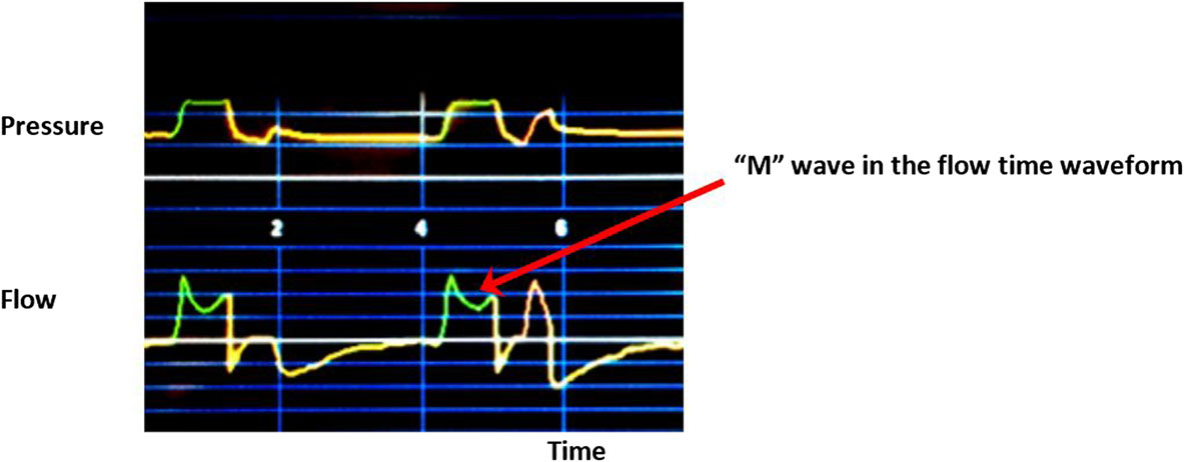
An “M” wave is seen in the flow-time waveform (flow starvation), followed by a spontaneous (pressure-supported) breath

In participants assigned to MBV, dyssynchrony will often cause large spikes in the elastance recordings. The CURE soft algorithm does not account for patient breathing efforts and “sees” inspiratory effort as a rapid reduction in lung elastance [32, 33]. In contrast, coughing, breath-holding, and other dyssynchronous efforts may cause an apparent increase in elastance [34]. Figure 2 shows an example of ventilator dyssynchrony in a pressure-controlled mode. Dyssynchrony may be seen as negative deflections (“M” waves) in the flow-time waveform, as shown in Fig. 2. In contrast the airway pressure may only be changed minimally by patient effort.

It is important to exclude reversible mechanical causes that might lead to patient distress and ventilator dyssynchrony. Endobronchial intubation, obstruction of a major bronchus or pneumothorax should be excluded.

Usually ventilator dyssynchrony can be managed by increasing sedation. However, in many cases it may be preferable to use intermittent muscle relaxants to fully control ventilation. It also may be helpful to trial the patient on assisted spontaneous breathing (ASB) to improve ventilation synchrony, if PEEP is ≤ 10 cmH_2_O and the FiO_2_ is ≤ 40%. However, caution should be exercised, lest the patient become exhausted (see “Weaning”).

## Weaning assessment

These guidelines are a pragmatic and consistent way to transition patients to ASB. Weaning is challenging and difficult to protocolise because there are many different factors to consider. For this reason, the weaning process is typically determined by clinical judgement. However, the guidelines presented here are set to ensure consistency of care.

ASB is considered when the participant’s condition is improving. They should preferably be afebrile, have resolution of the underlying processes that led to their ICU admission and improving gas exchange. They should have improving muscle strength, decreasing sedation requirements with an improving Glasgow Coma Score (GCS) and Richmond Agitation Sedation Score (RASS) between −3 and +1. Generally, the FiO_2_ should be ≤ 40% and PEEP ≤ 10 cmH_2_O.

If the following are substantially present, then participants may be transitioned to ASB:

1. Improving condition
2. Minute ventilation acceptable (VE) ≤ 0.2 L/kg
3. FiO_2_ ≤ 40%
4. SpO_2_ 93–95%
5. pH ≥ 7.3
6. Heart rate ≤ 120 beats/min
7. Low vasoactive drug requirements (noradrenaline + adrenaline ≤ 10 mcg/min)

The following instructions or recommendations are used to guide transition to ASB:

1. Set mandatory respiratory rate (RR) at ≤ 10 cmH_2_O.
2. Set pressure support (PS) at 10–15 cm cmH_2_O.
3. Consider PEEP level.
  - Generally, keep PEEP at ≤ 10 cmH_2_O.
  - PEEP maybe up to 15 cmH_2_O in obese participants or when the chest wall or abdominal elastance is increased.
4. Monitor RR, HR and SpO_2_ over the next 30 min. If there is significant increase in distress, desaturation, or an increased oxygen requirement, the participant is reverted back to the previous controlled ventilation mode.
5. If there is no significant deterioration, change the ventilation mode to ASB.
6. If the participant is comfortable, you may reduce PEEP and PS after 12 h. Titrate PEEP and PS as clinically indicated by ≤ 2 cmH_2_O PEEP; PS should remain at ≥ 5 cmH_2_O. Reductions in PEEP and PS should be generally made between 0800 and 1800 hours.

### Observations during ASB

The following observations should be made:

1. Check RR, HR and SpO_2_ every 2 h:
  - If there are increases in heart rate, agitation, delirium, respiratory distress, minor desaturations, or increasing oxygen requirements (ΔFiO_2_ ≥ 10% or FiO_2_ ≥ 50%), check the patient and the ventilator;
    i. Exclude patient conditions, e.g. endobronchial intubation, mucous plugging, pneumothorax etc.;
    ii. Optimise analgesia/sedation and patient position;
    iii. Consider increasing
      Pressure support up to 15 cm;
      Expiratory time for triggering (e.g. adjusting E sens up to 50% on the PB840 ventilator);
      Triggering sensitivity;
  - If the FiO_2_ is ≥ 50% or the FiO_2_ has increased by ≥ 10% in the previous 2 h, consider adjusting PEEP up (or down) by ≤ 2 cmH_2_O. Consider repositioning the patient and optimising sedation;
  - If secretions are obstructing large airways, or there are significant regions of consolidation, these conditions are unlikely to respond to increases in PEEP and increases in PEEP may impair oxygenation. Thus, if the PEEP is > 10 cmH_2_O, consideration should be given to reducing it;
  - If the aforementioned measures do not improve oxygenation, re-sedate and revert back to the previous controlled ventilation mode (Bi-Level or PC-SIMV+ or equivalent);
  - If the oxygenation has not improved after 12 h on controlled ventilation, or there is an unanticipated new problem causing deterioration, the participant should return to their previously assigned ventilation arm (MBV or SPV), e.g.:
    iv. New lung injury/de-recruitment/aspiration/sepsis.
    v. Haemodynamic instability.
    vi. Need to return to the operating room or to undergo invasive procedure.
2. If there is continual improvement, proceed towards separation from mechanical ventilation (extubation or continuous positive airway pressure (CPAP) via a tracheostomy).

## Patient enrolment and data management

A simplified flow chart of the patient enrolment process can be found in Additional file 1. All study data, including ventilation data, patient and family/friend consent, SAE reports and other documentation will be stored in a repository.

### Consolidated standards of reporting trials (CONSORT)

**Fig. 3:**
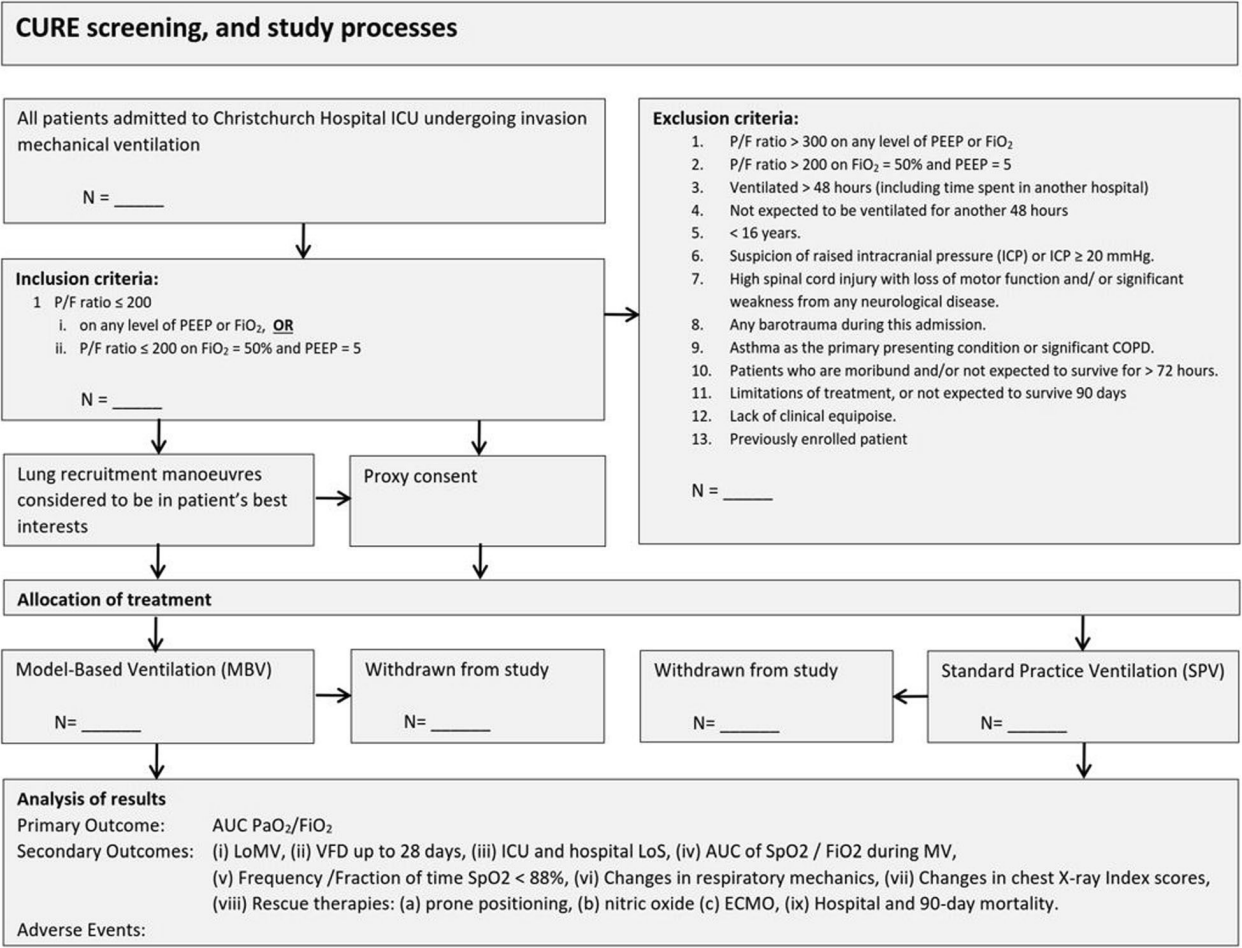
Consolidated standards of reporting trials (CONSORT) diagram for the CURE randomized controlled trial (RCT). P/F, partial pressure of arterial oxygen/fraction of inspired oxygen; PEEP, positive end-expiratory pressure; FiO_2_, fraction of inspired oxygen; COPD, chronic obstructive pulmonary disease; PAO_2_, partial pressure of arterial oxygen; LoMV, length of time of mechanical ventilation; VFD, ventilator-free days; SPO_2_, oxygen saturation; ECMO, extracorporeal membrane oxygenation

Figure 3 shows the CONSORT diagram for the CURE RCT.

### Data acquisition and management

Patients recruited into the CURE RCT will have the following data collected.

#### Patient demographic and history

The following data will be collected:

1. Patient gender, height, weight and ethnicity
2. Primary patient diagnosis contributing to ARDS or impaired lung function
3. Secondary patient diagnosis contributing to ARDS or impaired lung function
4. Relevant past medical history, e.g. smoking, medication, cardiovascular disease
5. Chest x-ray score derived by the Murray Index [35]

#### Patient mechanical ventilation data

Data on patient airway pressure and flow generated from the mechanical ventilator will be recorded using the CURE software (CURE Soft.) provided with the RCT. The patient data are backed up regularly to external storage with encryption applied using VeraCrypt encryption software [36].

#### Other patient information

The following information will be collected:

1. Demographic data
  - Age, weight, height
  - Ethnicity
  - Acute Physiology and Chronic Health Evaluation (APACHE) II and IIIJ, Simplified Acute Physiology Score (SAPS) II, Australian and New Zealand Risk of Death (ANZROD) from the ANZICS CORE database
2. Waveform data from physiological monitors including:
  - SpO_2_ - used to detect desaturation events
  - Blood pressure
  - Heart rate
  - End-tidal CO_2_
3. Arterial blood gases:
  - Measured at least twice daily
  - Before and after any RM_Max_ or PUMP
4. Routine biochemical analysis (up to daily)
  - Electrolytes, urea, creatinine, glucose, liver tests
  - Full blood count
5. Patient’s findings on radiology (chest x-ray and computed tomography (CT))
6. Patient position during MV (to account for data variation resulting from changes in patient position)
7. Rescue therapies: (i) prone positioning, (ii) nitric oxide (iii) ECMO
8. Length of time of mechanical ventilation (LoMV) or ventilation-free days
9. Amount of sedation - to account for possible data variation resulting from use of different sedatives
10. Duration of ICU stay
11. Frequency and duration of renal support therapies
12. All causes of ICU, hospital and 90-day mortality

#### Blood samples

No person or authority will have access to the participant’s blood. The blood samples are not stored. They are discarded and incinerated as soon as practicable, in accordance with NZS 4304:2002 “Healthcare Waste Management”.

#### Data management

All CURE RCT data will be stored at the University of Canterbury (UC). All paper forms (patient sheets, consent forms, etc.) will be scanned and stored at the UC. All electronic data will be stored in double-encrypted repository and only the participating researchers have access to it. Currently there are no plans for sharing the data, but if requested, data may be shared. Participants in the study can request their copy of data.

The data will be backed up weekly and again, once the participant has finished the trial and left the hospital. This task will only be performed by the participating researchers. Any protocol variations will be followed up and noted. The CURE RCT will store data for 20 years.

### Study outcome

The trial will utilise a primary composite end point incorporating the AUC of the P/F ratio over the period of MV. Every participant in the intervention (MBV) group is compared with every participant in the control (SPV) group. Test statistics will be calculated using the one-sided Wilcoxon rank sum test at alpha of 0.05. If results show no statistically significant difference between the intervention and control, it will result in the rejection of the intervention treatment as a standard of care and thus, the secondary clinical outcome assessments will include the number of desaturation events measured as peripheral capillary oxygen saturation < 90%, LoMV, VFD for 28 days, the quality of mechanical ventilation care measured as the AUC of SpO_2_/FiO_2_ and chest x-ray index scores over time. The test statistic will be calculated using the one-sided Wilcoxon rank sum test at alpha of 0.05.

A difference in the primary outcome will show the impact of MBV compared to SPV. No difference would show that enhanced, model-based metrics of the patient-specific condition have no effect on patient-centred or clinical outcomes. Either outcome will yield clinical guidance.

### Sample size study

A Monte-Carlo simulation was performed to determine the sample size and determined that a minimum effective sample size of approximately 160 patients per arm is required to identify a 25% reduction in median LoMV, with 0.8 power, at a double-sided significance level of 5% [37].

### Stopping rule

A linear alpha spending approach will be used for early termination of the trial for safety. Linear alpha spending falls between a Pocok and O’Brien-Fleming boundary [38]. With analysis points of 50, 75, 100, 125 and 160 patients per arm, and assumed control group mortality of 0.2, the mortality difference required to stop the trial (Mortality_Intervention_ – Mortality_control_) at each analysis point respectively is 0.20, 0.16, 0.14, 0.12 and 0.10. This approach has cumulative α = 0.025.

### Safety, ethics and dissemination

Ethics approval has been filed with the New Zealand National Health and Disability Ethics Committee. The CURE RCT clinical protocol and data usage has been filed with the New Zealand South Regional Ethics Committee (reference number 14/STH/132). The CURE trial is also registered in the Australian New Zealand Clinical Trial Registry (ACTRN12614001069640).

All results and any subsequent analysis will be published and only the participating investigators will be authors. Currently there is no plan to share data with other organisations. The data collected in this study will also be used for future research.

### Adverse event (AE) and SAE reporting

(AEs are defined as any unexpected change in physiology in a study participant associated with either the RM_max_ or PUMP. This does not necessarily have to have a causal relationship with the aforementioned procedures. Typically, this would be an unexpected, non-life-threatening event, which rapidly resolves following simple corrective measures. For example, hypotension will occur in most participants undergoing an RMmax or PUMP. However, if the procedure had to be shortened or abandoned, but the participant recovered with simple corrective measures (e.g. temporarily increasing noradrenaline by ≥ 5 mcg/min) or giving > 500 ml fluid bolus) this would be recorded as an AE. It is very important these events are accurately recorded as risk factors for AEs that need to be defined when carrying out RMs (as seen in Additional file 3).

SAEs are defined as any untoward medical occurrence that (1) results in death; (2) is life-threatening; (3) prolongs hospitalisation or (4) results in disability or incapability. However, baseline mortality in the patients in intensive care who are enrolled in the trial will likely be high due to the critical illness necessitating admission to the ICU. Despite attempts at prevention, trial participants will frequently develop life-threatening organ failure(s) unrelated to study interventions. Events that are a part of the natural history of the primary disease process or expected complications of critical illness will not be reported as SAEs in this trial. Additionally, events already defined and reported as study outcomes, such as mortality or re-admission to the ICU, will not be labelled and reported separately as SAEs unless they are considered to be causally related to the study intervention or are otherwise of concern in the investigator’s judgement.

SAEs will be reported to the principal investigator within 24 h of any investigator becoming aware of the event. The minimum information to report includes:

- Patient trial identifier
- The nature of the event
- The time the event commenced and ceased
- An investigator’s opinion of the relationship between study involvement and the event (not related, unlikely, possibly, probably or definitely related)
- Whether treatment was required for the event and what treatment was administered

SAEs could include pneumothorax, hypotension leading to cardiac arrest, transient desaturation leading to severe or prolonged desaturation, tachycardia, bradycardia, arrhythmia, anaphylaxis and unintended protocol deviations.

In the unlikely event of a physical injury to the participant as a result of their participation in this study, they will be eligible to apply for compensation from the Accident Compensation Corporation (ACC) NZ within its limitations. If the participant’s family/friend have any questions about the ACC, they will be able to ask the researchers for more information before they agree to take part in this trial.

ACC cover is not automatic and their case will need to be assessed by the ACC according to the provisions of the 2001 Injury Prevention Rehabilitation and Compensation Act. If the claim is accepted by the ACC, the patient still might not receive compensation. This depends on a number of factors such as whether they are earners or non-earners. The ACC usually provides only partial reimbursement of costs and expenses and there may be no lump sum compensation payable. There is no cover for mental injury unless it is a result of physical injury. If your relative or friend has ACC cover, generally this will affect their right to sue the investigators.

### Data Monitoring Committee (DMC)

An independent DMC comprising experts in clinical trials, biostatistics and intensive care medicine is established before patient enrolment, to review all trial protocols and oversee and advise on this trial. The DMC will be forwarded a copy of all SAE reports as soon as they become available to the trial investigators. The DMC will review all SAE reports that they receive and report back to investigators if any further action is required.

### CURE RCT composition

The steering committee of the CURE RCT comprises the primary investigators Geoff Shaw, Geoff Chase, Chris Pretty and Yeong Shiong Chiew. The clinical data are collected by research nurses in the ICU and mechanical ventilation data and oxygenation (bedside monitor) data will be collected by researchers from the UC. All study data will be stored in the double-encrypted repository at UC. Data will be interpreted by participating researchers. The open and closed case interim reports will be compiled by Paul Docherty every 6 months and when 50 and 100 patients have been included. The DMC will have authority to continue or stop of the trial based on the interim reports.

## Discussion

Mechanical ventilation using PEEP set at minimum elastance has long been investigated in both experimental and clinical trials. These studies ranged from healthy patients under general anaesthesia to those with ARDS. However, only a few studies have investigated the clinical potential of PEEP set at minimum elastance. Recent studies by Pintado et al. [22] and Chiew et al. [23] have shown the potential and feasibility of ventilating patients using minimum elastance PEEP. However, setting PEEP based on elastance is problematic due to the increased need of muscle relaxants, protocol burden and potential contradictory findings [22, 23]. The pilot trial was also underpowered and thus, a larger clinical trial such as the CURE RCT is required to provide further insight and validate the potential benefit of optimising mechanical ventilation PEEP with model-based methods.

The CURE RCT implements a protocolised staircase PEEP recruitment manoeuvre together with novel computer software to calculate respiratory system elastance in real time. The computer software, CURE Soft [25], uses a single compartment lung model [39] together with other model-based approach [24, 40] to aid clinicians during PEEP selection. This process potentially reduces selected PEEP variability and provides more consistent clinical guidance.

There are several limitations of the CURE RCT design that should be noted. In particular, the RM is a double-staircase manoeuvre and is design specific. Studies have shown that not all patients benefit from RMs [41, 42], and the benefit of an RM is dependent on the patient-specific disease state, as well as the design of the RM. The double-staircase RM in this trial was designed to assess lung recruitment and provide consistent PEEP titration. It is noted that not all patients included in this study will necessarily demonstrate alveolar lung recruitment.

Another limitation worth noting is the control group clinical protocol. Clinically, there is relatively little consensus an optimal mechanical ventilation mode. Thus, the standard practice ventilation in the participating hospital relies on general approaches [2] and is highly variable between clinicians. There may be no equal comparator for a mechanical ventilation study resulting from this variability. Patients recruited to the CURE RCT will have the MV mode set to bi-level ventilation in both the intervention and the control group; it is debatable that bi-level ventilation may lack certain ventilation advantages. However, this procedure will reduce variability and provides the opportunity for fair comparison between groups.

In the participating ICU, there are > 700 patients per year requiring invasive MV; however, only an average of < 5 patients were diagnosed with ARDS as the primary diagnosis per year. ARDS is nearly always regarded as a complication of an acute process. One concern is that the desired sample size cannot be achieved. However, this number is also too small compared to reports [43, 44]. The small number may be due to changes in the ARDS definition [27, 45] and misdiagnosis [46]. Estenssoro et al. [46] report that misdiagnosis could occur due to delayed screening. Thus, in the CURE RCT, any patient requiring invasive mechanical ventilation is screened immediately, as per Villar et al. [27], whereby the P/F ratio is measured at PEEP = 5 cmH_2_O, and FiO_2_ = 50%. Equally, retrospective screening was also performed and identified > 200 patients eligible for the trial per year. Hence, a 3-year study is planned to achieve the target sample size at an estimated recruitment rate of 50%.

## Conclusion

Optimising patient-specific mechanical ventilator settings remains a huge clinical challenge due to patient disease variability, as well as clinical practice variability. Thus, there is a need for a method to provide consistent patient-specific treatment. The CURE RCT is the first single-centre large clinical RCT using model-based minimum elastance PEEP selection in mechanical ventilation. It provides a means to select patient-specific PEEP in a consistent fashion and patient outcomes are compared to current practice. The CURE RCT investigation group hope that the results from this trial will support the use of model-based methods to estimate optimal PEEP, and will serve as a platform to assess other patient-centred outcomes in future mechanical ventilation studies in ARDS/ALI.

## Trial status

The trial has not started recruiting yet. The trial is estimated to start in December 2019 in Christchurch Hospital Intensive Care Unit. The trial is estimated to be completed by May 2022. This is protocol version number 2.0, dated 20 August 2019.

## Supplementary information

**Additional file 1.** Screening process diagram.

**Additional file 2.** Sample consent and study information forms. (7Z 273 kb)

**Additional file 3.** Adverse events and serious adverse events reporting.

## Data Availability

Currently there are no plans to share any of the data.

## Abbreviations

AE: Adverse event
ALI: Acute lung injury
APACHE III: Acute physiology and chronic health evaluation III
ARDS: Acute respiratory distress syndrome
ASB: Assisted spontaneous breathing
CO_2_: Carbon dioxide
CT: Computed tomography
CURE: Clinical utilisation of respiratory elastance
DMC: Data monitoring committee
ECMO: Extra-corporeal membrane oxygenation
FiO_2_: Fraction of inspired oxygen
IBW: Ideal body weight
ICU: Intensive care unit
i.v.: Intravenous
LoMV: Length of mechanical ventilation
LoS: Length of stay
MAP: Mean arterial pressure
MaxRM: Maximum recruitment manoeuvre
MBV: Model-based mechanical ventilation
MV: Mechanical ventilation
P/F: Partial pressure of arterial oxygen/fraction of inspired oxygen
PaCO_2_: Partial pressure of arterial carbon dioxide
PaO_2_: Partial pressure of arterial oxygen
PEEP: Positive end-expiratory pressure
Pi: Peak inspiratory pressure
PUMP: Pressure adjustment and monitoring procedure
RCT: Randomised controlled trial
RM: Recruitment manoeuvre
RR: Respiratory rate
SAE: Serious adverse event
SIMV: Synchronous intermittent mandatory ventilation
SPO_2_: Oxygen saturation
SPV: Standard practice ventilation
VFD: Ventilator-free days (up to 28 days)
VILI: Ventilator-induced lung injury
V_T_: Tidal volume

## References

[1] Girard TD, Bernard GR (2007). Mechanical ventilation in ARDS: a state-of-the-art review. Chest.

[2] ARDS Network (2000). Ventilation with lower tidal volumes as compared with traditional tidal volumes for acute lung injury and the acute respiratory distress syndrome. N Eng J Med.

[3] Amato MB, Barbas CS, Medeiros DM, Magaldi RB, Schettino GP, Lorenzi-Filho G (1998). Effect of a protective-ventilation strategy on mortality in the acute respiratory distress syndrome. N Engl J Med.

[4] Brower RG, Lanken PN, MacIntyre N, Matthay MA, Morris A, Ancukiewicz M (2015). Higher versus lower positive end-expiratory pressures in patients with the acute respiratory distress syndrome. N Engl J Med.

[5] Villar J, Kacmarek RM, Perez-Mendez L, Aguirre-Jaime A (2006). A high positive end-expiratory pressure, low tidal volume ventilatory strategy improves outcome in persistent acute respiratory distress syndrome: a randomized, controlled trial. Crit Care Med.

[6] Meade MO, Cook DJ, Guyatt GH, Slutsky AS, Arabi YM, Cooper DJ (2008). Ventilation strategy using low tidal volumes, recruitment maneuvers, and high positive end-expiratory pressure for acute lung injury and acute respiratory distress syndrome: a randomized controlled trial. JAMA.

[7] Mercat A, Richard JM, Vielle B (2016). Positive end-expiratory pressure setting in adults with acute lung injury.

[8] Oba Y, Thameem DM, Zaza T (2009). High levels of PEEP may improve survival in acute respiratory distress syndrome: a meta-analysis. Respir Med.

[9] Briel M, Meade M, Mercat A, Brower RG, Talmor D, Walter SD (2010). Higher vs lower positive end-expiratory pressure in patients with acute lung injury and acute respiratory distress syndrome. J Am Med Assoc.

[10] Hickling KG, Henderson SJ, Jackson R (1990). Low mortality associated with low volume pressure limited ventilation with permissive hypercapnia in severe adult respiratory distress syndrome. Intensive Care Med.

[11] Gattinoni L, Carlesso E, Brazzi L, Caironi P (2010). Positive end-expiratory pressure. Curr Opin Crit Care.

[12] Guerin C (2011). The preventive role of higher PEEP in treating severely hypoxemic ARDS. Minerva Anestesiol.

[13] Thammanomai A, Hamakawa H, Bartolák-Suki E, Suki B (2013). Combined effects of ventilation mode and positive end-expiratory pressure on mechanics, gas exchange and the epithelium in mice with acute lung injury. PLoS One.

[14] Borges JB, Okamoto VN, Matos GFJ, Caramez MPR, Arantes PR, Barros F (2006). Reversibility of lung collapse and hypoxemia in early acute respiratory distress syndrome. Am J Respir Crit Care Med.

[15] de Matos GFJ, Stanzani F, Passos RH, Fontana MF, Albaladejo R, Caserta RE (2012). How large is the lung recruitability in early acute respiratory distress syndrome: a prospective case series of patients monitored by computed tomography. Crit Care.

[16] Malbouisson LM, Muller JC, Constantin JM, Lu Q, Puybasset L, Rouby JJ (2001). Computed tomography assessment of positive end-expiratory pressure-induced alveolar recruitment in patients with acute respiratory distress syndrome. Am J Respir Crit Care Med.

[17] Rose L, Presneil JJ, Johnston L, Nelson S, Cade JF (2009). Ventilation and weaning practices in Australia and New Zealand. Anaesth Intensive Care.

[18] Carvalho A, Jandre FC, Pino AV, Bozza FA, Salluh J, Rodrigues R (2007). Positive end-expiratory pressure at minimal respiratory elastance represents the best compromise between mechanical stress and lung aeration in oleic acid induced lung injury. Crit Care.

[19] Suarez-Sipmann F, Böhm SH, Tusman G, Pesch T, Thamm O, Reissmann H (2007). Use of dynamic compliance for open lung positive end-expiratory pressure titration in an experimental study. Crit Care Med.

[20] Lambermont B, Ghuysen A, Janssen N, Morimont P, Hartstein G, Gerard P (2008). Comparison of functional residual capacity and static compliance of the respiratory system during a positive end-expiratory pressure (PEEP) ramp procedure in an experimental model of acute respiratory distress syndrome. Crit Care.

[21] Suter PM, Fairley HB, Isenberg MD (1975). Optimum end-expiratory airway pressure in patients with acute pulmonary failure. N Engl J Med.

[22] Pintado M-C, de Pablo R, Trascasa M, Milicua J-M, Rogero S, Daguerre M (2013). Individualized PEEP setting in subjects with ARDS: a randomized controlled pilot study. Respir Care.

[23] Chiew YSW, Pretty CG, Shaw GM, Chiew YSW, Lambermont B, Desaive T (2015). Feasibility of titrating PEEP to minimum elastance for mechanically ventilated patients. Pilot Feasibility Stud.

[24] Chiew YS, Chase JG, Shaw GM, Sundaresan A, Desaive T (2011). Model-based PEEP optimisation in mechanical ventilation. Biomed Eng Online.

[25] Szlavecz A, Chiew YS, Redmond D, Beatson A, Glassenbury D, Corbett S (2014). The clinical utilisation of respiratory elastance software (CURE Soft): a bedside software for real-time respiratory mechanics monitoring and mechanical ventilation management. Biomed Eng Online.

[26] Definition TB, The ARDS Definition Task Force, Definition TB. Acute Respiratory Distress Syndrome. JAMA. 2012;307. 10.1001/jama.2012.5669. 10.1001/jama.2012.5669 22797452

[27] Villar J, Pérez-Méndez L, Blanco J, Añón JM, Blanch L, Belda J (2013). A universal definition of ARDS: the PaO2/FiO2 ratio under a standard ventilatory setting—a prospective, multicenter validation study. Intensive Care Med.

[28] Brochard L, Costa ELV, Schoenfeld DA, Ph D, Stewart TE, Briel M (2015). Driving pressure and survival in the acute respiratory distress syndrome. N Engl J Med.

[29] Brochard L, Slutsky A, Pesenti A (2017). Mechanical ventilation to minimize progression of lung injury in acute respiratory failure. Am J Respir Crit Care Med.

[30] Brochard L (2017). Ventilation-induced lung injury exists in spontaneously breathing patients with acute respiratory failure: Yes. Intensive Care Med.

[31] Redmond D, Chiew YS, Van Drunen E, Shaw GM, Chase JG (2014). A minimal algorithm for a minimal recruitment model-model estimation of alveoli opening pressure of an acute respiratory distress syndrome (ARDS) lung. Biomed Signal Process Control.

[32] Thille AW, Rodriguez P, Cabello B, Lellouche F, Brochard L (2006). Patient-ventilator asynchrony during assisted mechanical ventilation. Intensive Care Med.

[33] Carlucci A, Pisani L, Ceriana P, Malovini A, Nava S (2013). Patient-ventilator asynchronies: may the respiratory mechanics play a role?. Crit Care.

[34] De Wit M, Pedram S, Best AM, Epstein SK (2009). Observational study of patient-ventilator asynchrony and relationship to sedation level ☆. J Crit Care.

[35] Murray John F., Matthay Michael A., Luce John M., Flick Michael R. (1988). An Expanded Definition of the Adult Respiratory Distress Syndrome. American Review of Respiratory Disease.

[36] IDRIX, VeraCrypt c2013-2020. Available from: https://www.veracrypt.fr/en/Downloads.html. [Cited Dec 2019].

[37] Morton SE, Chiew YS, Pretty C, Moltchanova E, Scarrott C, Redmond D (2017). effective sample size estimation for a mechanical ventilation trial through monte-carlo simulation: length of mechanical ventilation and ventilator free days. Math Biosci.

[38] Fleming TR, Harrington DP, O’Brien PC (1984). Designs for group sequential tests. Control Clin Trials.

[39] Lucangelo U, Bernabè F, Blanch L (2007). Lung mechanics at the bedside: make it simple. Curr Opin Crit Care.

[40] Chiew YS, Pretty C, Docherty PD, Lambermont B, Shaw GM, Desaive T (2015). Time-varying respiratory system elastance: a physiological model for patients who are spontaneously breathing. PLoS One.

[41] Fan E, Wilcox ME, Brower RG, Stewart TE, Mehta S, Lapinsky SE (2008). Recruitment maneuvers for acute lung injury. Am J Respir Crit Care Med.

[42] Pelosi Paolo, de Abreu Marcelo, Rocco Patricia RM (2010). New and conventional strategies for lung recruitment in acute respiratory distress syndrome. Critical Care.

[43] Bersten AD, Edibam C, Hunt T, Moran J, The Australian and New Zealand Intensive Care Society Clinical Trials Group (2002). Incidence and mortality of acute lung injury and the acute respiratory distress syndrome in three Australian States. Am J Respir Crit Care Med.

[44] Rubenfeld GD, Caldwell E, Peabody E, Weaver J, Martin DP, et al. Incidence and outcomes of acute lung injury. 2012:1685–93. 10.1056/NEJMc053159. 10.1056/NEJMoa050333 16236739

[45] Bernard GR, Artigas A, Brigham KL, Carlet J, Falke K, Hudson L (1994). Report of the American-European consensus conference on ARDS: definitions, mechanisms, relevant outcomes and clinical trial coordination. Intensive Care Med.

[46] Estenssoro E, Dubin A, Laffaire E, Canales HS, Sáenz G, Moseinco M (2003). Impact of positive end-expiratory pressure on the definition of acute respiratory distress syndrome. Intensive Care Med.

