# Supplementary material for: Model-based PEEP titration versus standard practice in mechanical ventilation: a randomised controlled trial
Source: Trials. 2020 Feb 1;21:130. doi: 10.1186/s13063-019-4035-7 (PMC6995650; doi:10.1186/s13063-019-4035-7)
Supplement: Supplementary file 1 — Additional file 1. Screening process diagram. [file 13063_2019_4035_MOESM1_ESM.pdf]

CURE screening, and study processes

All patients admitted to Christchurch Hospital ICU undergoing invasion mechanical ventilation

N = ______

Inclusion criteria:

1 P/F ratio ≤ 200

i. on any level of PEEP or FiO_2, **OR**

ii. P/F ratio ≤ 200 on FiO_2 = 50% and PEEP = 5

N = ______

Exclusion criteria:

1. P/F ratio > 300 on any level of PEEP or FiO_2
2. P/F ratio > 200 on FiO_2 = 50% and PEEP = 5
3. Ventilated > 48 hours (including time spent in another hospital)
4. Not expected to be ventilated for another 48 hours
5. < 16 years.
6. Suspicion of raised intracranial pressure (ICP) or ICP ≥ 20 mmHg.
7. High spinal cord injury with loss of motor function and/ or significant weakness from any neurological disease.
8. Any barotrauma during this admission.
9. Asthma as the primary presenting condition or significant COPD.
10. Patients who are moribund and/or not expected to survive for > 72 hours.
11. Limitations of treatment, or not expected to survive 90 days
12. Lack of clinical equipoise.
13. Previously enrolled patient

N = ______

Lung recruitment manoeuvres considered to be in patient's best interests

Proxy consent

Allocation of treatment

Model-Based Ventilation (MBV)

N= _______

Withdrawn from study

N= _______

Withdrawn from study

N= _______

Standard Practice Ventilation (SPV)

N= _______

Analysis of results

Primary Outcome: AUC PaO_2/FiO_2

Secondary Outcomes: (i) LoMV, (ii) VFD up to 28 days, (iii) ICU and hospital LoS, (iv) AUC of SpO2 / FiO2 during MV, (v) Frequency /Fraction of time SpO2 < 88%, (vi) Changes in respiratory mechanics, (vii) Changes in chest X-ray Index scores, (viii) Rescue therapies: (a) prone positioning, (b) nitric oxide (c) ECMO, (ix) Hospital and 90-day mortality.

Adverse Events:
