# Supplementary material for: Model-based PEEP titration versus standard practice in mechanical ventilation: a randomised controlled trial
Source: Trials. 2020 Feb 1;21:130. doi: 10.1186/s13063-019-4035-7 (PMC6995650; doi:10.1186/s13063-019-4035-7)
Supplement: Supplementary file 3 — Additional file 3. Adverse events and serious adverse events reporting. [file 13063_2019_4035_MOESM3_ESM.docx]

**Adverse Events and Serious Adverse Events Reporting**

The following is based on the consensus statement for reporting adverse events (or experiences) Note for Guidance on Clinical Safety Data Management: Definitions and Standards for Expedited Reporting (CPMP/ICH/377/95)

<https://www.ema.europa.eu/en/documents/scientific-guideline/international-conference-harmonisation-technical-requirements-registration-pharmaceuticals-human-use_en-15.pdf> (accessed 14 November 2019)

**1 Adverse Event (or Adverse Experience)**

*“Any untoward medical occurrence in a patient or clinical investigation subject administered a pharmaceutical product and which does not necessarily have to have a causal relationship with this treatment.”*

With regard to the recruitment intervention, an adverse event (AE) is defined as:

Any unexpected change in physiology in a study participant associated with either the maximum recruitment manoeuvre (RM_max_) or PEEP adjustment and monitoring procedure (PUMP). This does not necessarily have to have a causal relationship with the above procedures. Typically this would be an unexpected, non-life threatening event, which rapidly resolves following simple corrective measures. For example, hypotension will occur in most participants under-going an RM_max_, or PUMP. However if the procedure had to be shortened or abandoned, but the participant recovered with simple corrective measures (e.g. temporarily increasing noradrenaline ≥ 5 mcg / min) or giving more than a 500 ml fluid bolus) this would be recorded as an Adverse Event (AE). It is very important these events are accurately recorded as risk factors for AEs need to be defined when carrying out RMs.

**2 Serious Adverse Event**

*“A serious adverse event (experience) or reaction is any untoward medical*

*occurrence that at any dose:*

- *results in death,*
- *is life-threatening,*
- *requires inpatient hospitalisation or prolongation of existing*
- *hospitalisation,*
- *results in persistent or significant disability/incapacity”*

With regard to the recruitment intervention, a serious adverse event (SAE) is defined as:

Any immediate life-threatening unexpected change in physiology in a study participant associated with either the maximum recruitment manoeuvre (RM_max_) or PEEP adjustment and monitoring procedure (PUMP). This does not necessarily have to have a causal relationship with the above procedures. This would be an unexpected life threatening event, which does not rapidly resolve following simple corrective measures. For example, severe hypotension or cardiac arrest /pulseless electrical activity in a participant under-going an RM_max_, or PUMP, which required significant resuscitative measures. In addition, evidence of barotrauma (e.g. pneumothorax) at any time is regarded as an SAE. See table for other definitions. All events considered to be SAE must be immediately notified to the research team.

It is very important AEs and SAEs are accurately recorded as risk factors for carrying out recruitment manoeuvres need to be defined.

**Adverse Event (AE) Reporting Form**

**Patient Trial Number**

|  |
| --- |

**Patient conditions (list)**

|  |
| --- |

**AE Types**

|  | Hypoxaemia |  | Respiratory dyssynchrony |
| --- | --- | --- | --- |
|  | Hypotension |  | Other: |
|  | Agitation / distress |  |  |

**AE Description and Treatment:**

| (Please record any particular treatment given) |
| --- |

**Principal/ Investigator Suspected Relationship of AE to Study Treatment:**

|  | Not Related |
| --- | --- |
|  | Unlikely |
|  | Possible |
|  | Probably |
|  | Definitely |

SAE Onset Date: ______/______/__________ (DD/ MM/ YYYY)

Action Taken with Study Protocol:

|  | None |
| --- | --- |
|  | Recruitment Manoeuvre or PUMP manoeuvre modified due to hypotension or hypoxaemia. Specify what was done: |

Investigator’s Signature:

Investigator’s Name:

Date: ______/______/__________

**Serious Adverse Event Reporting Form**

**Patient Trial Number**

|  |
| --- |

**Patient conditions (list)**

|  |
| --- |

**SAE Types**

|  | Death |  | Result in disability and incapability |
| --- | --- | --- | --- |
|  | Life Threatening |  | Other: |
|  | Prolongation of Hospitalisation |  |  |

**SAE Description and Treatment:**

| (Please record any particular treatment given) |
| --- |

**Principal/ Investigator Suspected Relationship of SAE to Study Treatment:**

|  | Not Related |
| --- | --- |
|  | Unlikely |
|  | Possible |
|  | Probably |
|  | Definitely |

SAE Onset Date: ______/______/__________ (DD/ MM/ YYYY)

SAE Resolution Date: ______/______/__________ (DD/ MM/ YYYY)

Death Date: ______/______/__________ (DD/ MM/ YYYY)

**Outcomes:**

|  | Unknown/  Lost to Follow Up |  |  | |
| --- | --- | --- | --- | --- |
|  | Unresolved |  |  | |
|  | Resolved |  |  | |
|  | Resolved with Sequels | Please Specify |  | |
|  | Death | Cause of Death |  | |
|  |  | Autopsy |  | Report Attached |
|  |  |  |  | Not Done |

Action Taken with Study Protocol:

|  | None |
| --- | --- |
|  | Recruitment Manoeuvre or PUMP manoeuvre modified due to hypotension or hypoxaemia. Specify what was done: |
|  | Termination of Recruitment Manoeuvre or PUMP due to unexpected severe hypotension +/- cardiovascular compromise, or severe +/- prolonged hypoxaemia  Specify what was done: |

Additional Notes:

|  |
| --- |

Investigator’s Signature:

Investigator’s Name:

Date: ______/______/__________
